# Approaches to studying predict academic performance in undergraduate occupational therapy students: a cross-cultural study

**DOI:** 10.1186/s12909-017-0914-3

**Published:** 2017-05-02

**Authors:** Tore Bonsaksen, Ted Brown, Hua Beng Lim, Kenneth Fong

**Affiliations:** 1 Department of Occupational Therapy, Prosthetics and Orthotics, Faculty of Health Sciences, Oslo and Akershus University College of Applied Sciences, PO Box 4, St. Olavs Plass, Oslo, 0130 Norway; 2 Faculty of Health Studies, VID Specialized University, Sandnes, Norway; 3 Department of Occupational Therapy, School of Primary Health Care, Faculty of Medicine, Nursing and Health Sciences, Monash University – Peninsula Campus, Frankston, VIC Australia; 4 Occupational Therapy, School of Health Sciences (Allied Health), Nanyang Polytechnic, Singapore, Singapore; 5 Department of Rehabilitation Sciences, Hong Kong Polytechnic University, Hong Kong, Hong Kong

**Keywords:** Academic performance, Cross-cultural study, Grade point average, Higher education, Occupational therapy, Students

## Abstract

**Background:**

Learning outcomes may be a result of several factors including the learning environment, students’ predispositions, study efforts, cultural factors and approaches towards studying. This study examined the influence of demographic variables, education-related factors, and approaches to studying on occupational therapy students’ Grade Point Average (GPA).

**Methods:**

Undergraduate occupational therapy students (*n* = 712) from four countries completed the Approaches and Study Skills Inventory for Students (ASSIST). Demographic background, education-related factors, and ASSIST scores were used in a hierarchical linear regression analysis to predict the students’ GPA.

**Results:**

Being older, female and more time engaged in self-study activities were associated with higher GPA among the students. In addition, five ASSIST subscales predicted higher GPA: higher scores on ‘seeking meaning’, ‘achieving’, and ‘lack of purpose’, and lower scores on ‘time management’ and ‘fear of failure’. The full model accounted for 9.6% of the variance related to the occupational therapy students’ GPA.

**Conclusions:**

To improve academic performance among occupational therapy students, it appears important to increase their personal search for meaning and motivation for achievement, and to reduce their fear of failure. The results should be interpreted with caution due to small effect sizes and a modest amount of variance explained by the regression model, and further research on predictors of academic performance is required.

## Background

To support students to achieve success in the higher education system, one needs to have an understanding of the factors that influence their learning process – and in turn, their learning outcomes. A model of students’ learning in higher education was introduced by Biggs in 1987 [1], often referred to as the 3P model of learning: *presage, process,* and *product*. The *presage* factors are related to the students’ personal background, in essence, their sociodemographic characteristics and readiness for intellectual inquiry and understanding. Presage also consists of the situational context in which the learning takes place, which includes the particular field of study and its traditions, the frequently used teaching and assessment forms, and the time spent engaging with the relevant tasks. In short, the presage factors are those that constitute the background and context for the learning experience [1].

The learning *process*, in contrast, involves *how* the students engage with study content. Following the influential work by Marton and Säljö [2] and later contributions of Entwistle and colleagues [3–8], one important line of inquiry related to students’ learning processes used the analytic categories of deep, surface, and strategic learning as a point of departure. Deep learners try to connect new to old ideas and seek to understand the meanings and possible applications of the study curriculum and materials. The motivation of deep learners is to gain a more comprehensive understanding of the subject matter. In contrast, students engaged with surface learning try to remember factual content and ensure that they go through the pre-planned syllabus. This often results in rote learning, as the motivation of surface learners is not to improve their understanding, but rather to avoid failure [9]. Students engaged with the third category, strategic learning, are oriented towards achievement on assessments, and pay much attention to the organizing and management of their study efforts in order to meet this end [8].

The *product* or outcome of the learning process related to Biggs' [1] 3P Model of Learning is generally considered to be related to both *presage* and *process* – the learning outcome is seen as a result of the interplay between student characteristics, the learning environment, and the way students engage with the content [7]. In consideration of the varied and complex tasks and situations an occupational therapist may face in his/her practice, the learning outcomes of occupational therapy students should mirror this variation and complexity [10]. Occupational therapy students need to demonstrate the skills relevant for hands-on clinical practice, but also need to demonstrate that they can apply relevant concepts and theories to a variety of clinical situations. Without conceptual frameworks to guide their application, clinical skills may not be applied in the most appropriate or efficient manner [11, 12]. Thus, learning outcomes for occupational therapy students need to include the academic aspect, and not just focus on clinical skills. In this study, we focused on the academic aspect of the students’ performance.

When considering associations between the *presage* factors and students’ academic performance, students who are older have been reported to achieve better academic outcomes, compared to younger students [13]. A mediation effect has been suggested to explain this finding: the positive effect of higher age may in part rely on the sources of motivation and the ways that older and more experienced students relate to study content - that is, they tend to be more intrinsically motivated and are more inclined to use deep learning strategies [13–16]. A sophisticated model was tested in a recent study of Dutch medical students [17], where a measure of balanced motivation predicted the use of good study strategies and more study efforts, in turn predicting better academic performance. In a systematic review and meta-analysis [18], where the researchers had screened 7167 articles published between 1997 and 2010 for relevance, female gender was found to be associated with better academic performance, compared to males, but research results from various educational fields are contradictory on this matter [13, 19–22]. As suggested from the literature, the effect of gender may also depend on the field of study and how students approach studying.

When considering *process*, therefore, deep and strategic approaches to studying are generally associated with better learning outcomes when compared with surface approaches [17, 18, 20, 23, 24]. Learning outcomes used in previous research with healthcare students include higher Grade Point Average (GPA) [25–29], better clinical examination outcomes, and better performance on fieldwork placements [30, 31]. There is, however, little research specifically focused on occupational therapy students’ approaches to study. Svidén [32] used a phenomenographic approach involving 36 occupational therapy students and found that deep and surface approaches to studying, as described by Entwistle and Ramsden [8], were applicable to the students’ descriptions of their learning process. Chapman, Watson and Adams [33] followed-up occupational therapy students from the United Kingdom (UK) (*n* = 138) and Bangladesh (*n* = 46) over a three year period of their study. They determined that the Bangladeshi students’ use of deep and surface approaches both exceeded the respective levels shown among the British students. A cross-cultural study published recently showed similarities as well as differences in learning approaches between undergraduate occupational therapy students from Australia, Norway, Hong Kong and Singapore [34].

In summary, there is much evidence to suggest that different approaches to studying have different impacts in terms of learning outcomes and subsequent academic success for students enrolled in higher education courses. However, research on approaches to studying among occupational therapy students is sparse and has mostly been conducted with relatively small and culturally homogenous samples. Moreover, to our knowledge, researchers in the field of occupational therapy education have not employed the current version of the Approaches and Study Skills Inventory for Students (ASSIST) [3], including its three main dimensions and 13 subscales. Thus, the more specific aspects of the approaches to studying, as measured with the ASSIST subscales, have not been subject to prior investigation in our field. The current study seeks to provide further insights into approaches to studying, and their associations with academic performance, in a large cross-cultural sample of undergraduate occupational therapy students. In light of the evidence provided from previous studies, it was hypothesized that higher scores on the ASSIST subscales related to the ‘deep’ and the ‘strategic’ approaches were associated with higher GPA among the students. Conversely, it was hypothesized that higher scores on the subscales related to the ‘surface’ approach were associated with lower GPA among the students.

### Study aim

The aim of the current study was to examine whether approaches to studying were predictive of undergraduate occupational therapy students’ academic success, as measured with their GPA, after controlling for demographic and education-related variables.

## Methods

### Design and setting of the study

A cross-sectional design study that included students from four different countries as participants was utilised. Prior to this study being conducted, the four university education programmes already had an established research collaboration that they wanted to expand on, and this factor determined the scope of the study. The occupational therapy education in Australia and Hong Kong is four years full time while the Singapore and Norwegian education is three years full time. All education programmes were at the undergraduate level. The curricula have some similarities, particularly in the first study year, in that they all adopt a traditional teaching approach where foundational subjects such as anatomy, physiology, psychology, and occupational therapy theories are introduced. The courses become more different from one another from the second year: from that point, the Australian and Norwegian students are more involved in scenario-based approach to teaching and learning, while the curriculum in Hong Kong and Singapore continues with a traditional didactic approach to teaching. All four education programmes meet the standards set by the World Federation of Occupational Therapists.

### Participants and recruitment

The inclusion criteria for the study were: 1) students enrolled in the undergraduate occupational therapy course of the education programme involved in the study; and 2) students provided informed consent to participate in the study. Sample size was not specified prior to the study. The researchers wanted an inclusive approach to study participation, as participation in research is sometimes considered an interesting and desired experience among students. However, according to Tabachnick and Fidell [35], a necessary sample size to be used in a multivariate analysis with 17 independent variables (as performed in the current study) would be approximately 200 participants. A non-teaching member of staff distributed the questionnaires to students during breaks in classrooms. The data were collected in 2015.

### Measurement

The outcome variable in the study was the students’ academic performance, operationalized as their current GPA at the time of the data collection. In Australia, Hong Kong, and Singapore, GPA scores were derived from the following exam results: ≤ 49% = 1, 50–59% = 2, 60–69% = 3, 70–79% = 4, 80–89% = 5, and ≥ 90% = 6. In Norway, GPA scores were based on the qualitative descriptors related to the students’ exam grades [36]: fail = 1, sufficient = 5, satisfactory = 3, good = 4, very good = 5, and excellent = 6.

Data on students’ approaches to studying was obtained from the Approaches and Study Skills Inventory for Students (ASSIST). The ASSIST has three sections, including a 6-item questionnaire of students’ conceptions of studying (section A), a 52-item questionnaire of students’ approaches to studying (section B), and an 8-item questionnaire of student preferences for teaching. In this study, we followed Entwistle and McCune’s [4] recommendation to use only the 52-item questionnaire (section B) instead of the whole instrument. The English version of the ASSIST [3] was used with the students studying in Australia, Hong Kong and Singapore, whereas the Norwegian students completed the Norwegian version of the instrument [37].

Factor analysis has confirmed that the ASSIST items can be meaningfully organized as three main factors, namely the *deep*, *strategic*, and *surface* approaches [6, 38, 39]. Each of the main factors consists of several subscales. The deep approach consists of the subscales ‘seeking meaning’, ‘relating ideas’, ‘use of evidence’, and ‘interest in ideas’. The strategic approach consists of ‘organized study’, ‘time management’, ‘alertness to assessment demands’, ‘achieving’, and ‘monitoring effectiveness’. The surface approach consists of ‘lack of purpose’, ‘unrelated memorizing’, ‘syllabus-bound’, and ‘fear of failure’.

The English version of the ASSIST scales has been shown to possess good internal consistency (Cronbach’s α ranging 0.61–0.88) when used with students in different academic and professional areas [21, 25, 38–40]. With the Norwegian version of the ASSIST [37], the same three latent factors have been found, and satisfactory measures of internal consistency has been established for each of them (Cronbach’s α ranging 0.70–0.81). In addition to the ASSIST, information regarding demographics (age and gender) and education (time spent on self-study and prior higher education) was collected using a brief questionnaire.

### Data analysis

All data were entered into the computer program IBM SPSS [41]. Descriptive analyses were performed on all variables using means (M), standard deviations (SD), frequencies and percentages as appropriate. Hierarchical linear regression analysis was used to assess the amount of variance in the participants’ GPA that was explained by demographic and education-related variables and by the ASSIST subscale scores. The analysis also assessed the strength of the associations between each of the independent variables and GPA among the participants. In the first block of the model, demographic and education-related variables were included: (1) age, gender, time spent on relevant self-study activities (average hours during a normal week) and previous higher education (yes or no). In the second block, the subscales belonging to the deep approach were included: (2) seeking meaning, relating ideas, use of evidence, and interest in ideas. The third block consisted of the subscales belonging to the strategic approach: (3) organized study, time management, alertness to assessment, achieving, and monitoring effectiveness. The fourth block included the subscales belonging to the surface approach: (4) lack of purpose, unrelated memorizing, syllabus-bound, and fear of failure. The level of statistical significance was set at *p* < 0.05.

## Results

### Participants

Seven hundred and twelve students (*n* = 376 from Australia, *n* = 109 from Hong Kong, *n* = 160 from Norway, and *n* = 67 from Singapore) completed the questionnaire. The Australian participants included students from all four years of study (first year *n* = 170, second year *n* = 77, third year *n* = 73, and fourth year *n* = 56). The Norwegian participants included all three year levels (first year *n* = 57, second year *n* = 50, and third year *n* = 53). The participants from Hong Kong were predominantly in their first and third year of studies (first year *n* = 37, second year *n* = 5, and third year *n* = 67). From Singapore, only first year students participated in the study (*n* = 67).

The majority of the students were in the age group 20–24 years (*n* = 416, 58.4%) and 86.8% of the sample was under the age of 25. Female students were also in vast majority (*n* = 602, 84.6%). Two hundred and sixty two students (36.8%) had higher education experience prior to enrolment in the occupational therapy program. On average, the participants reported that they spent 12.7 h (*SD* = 8.2 h) engaged in relevant self-study activities during a typical week. The sample mean of the GPA was 3.95 (*SD* = 1.02), indicating a ‘good’ level of GPA in the sample. The demographic characteristics of the study participants are displayed in Table 1.

**Table 1:** Characteristics of the study participants

Characteristics	Country of study				
	Australia (*n* = 376)	Hong Kong (*n* = 109)	Norway^a^ (*n* = 160)	Singapore^b^ (*n* = 67)	Total sample (*n* = 712)
Age group (years)	*n* (%)	*n* (%)	*n* (%)	*n* (%)	*n* (%)
15–19	125 (33.2)	31 (28.4)	6 (3.8)	39 (58.2)	201 (28.2)
20–24	214 (56.9)	69 (63.3)	107 (66.9)	26 (38.8)	416 (58.4)
25–29	16 (4.3)	8 (7.3)	29 (18.1)	1 (1.5)	54 (7.6)
30–35	8 (2.1)	0 (0.0)	10 (6.3)	1 (1.5)	19 (2.7)
36–39	8 (2.1)	1 (0.9)	4 (2.5)	0 (0.0)	13 (1.8)
>40	5 (1.3)	0 (0.0)	3 (1.9)	0 (0.0)	8 (1.1)
Gender					
Male	44 (11.7)	83 (76.1)	34 (21.3)	5 (7.5)	109 (15.3)
Female	332 (88.3)	26 (23.9)	126 (78.8)	61 (91.0)	602 (84.6)
Prior education					
Yes	162 (43.1)	27 (24.8)	70 (43.8)	3 (4.5)	262 (36.8)
No	214 (56.9)	82 (75.2)	90 (56.3)	64 (95.5)	450 (63.2)
	*M* (*SD*)	*M* (*SD*)	*M* (*SD*)	*M* (*SD*)	*M* (*SD*)
Self-study	13.5 (8.8)	11.8 (7.4)	9.6 (5.4)	17.4 (8.4)	12.7 (8.2)
GPA	3.7 (0.9)	4.5 (1.2)	4.1 (1.2)	4.2 (1.1)	4.0 (1.0)

*n* = number of participants *M* Mean, *SD* Standard Deviation. Self-study is reported as the number of hours engaged in self-studying during a typical week. GPA is reported on a 1–6 scale, where 1 = fail and 6 = excellent

^a^The data from Norway included one missing value on the age variable

^b^ The data from Singapore included one missing value on the gender variable

### ASSIST scores

The mean ASSIST category and subscale scores for the total sample and each of the four countries are reported in Table 2.

**Table 2:** The participants’ approaches to studying

ASSIST category	ASSIST subscales	Country of study				
		Australia (*n* = 376)	Hong Kong (*n* = 109)	Norway (*n* = 160)	Singapore (*n* = 67)	Total sample (*n* = 712)
		*M* (*SD*)	*M* (*SD*)	*M* (*SD*)	*M* (*SD*)	*M* (*SD*)
Deep approach to studying		55.90 (8.68)	57.56 (6.47)	57.55 (8.33)	57.70 (8.00)	56.68 (8.26)
	Seeking meaning	13.35 (2.52)	14.56 (2.15)	14.71 (2.40)	14.69 (2.34)	13.96 (2.51)
	Relating ideas	14.05 (2.99)	14.38 (2.00)	14.03 (2.81)	14.18 (2.89)	14.10 (2.81)
	Use of evidence	14.15 (2.97)	14.53 (2.08)	14.26 (2.61)	14.94 (2.30)	14.31 (2.70)
	Interest in ideas	14.35 (2.56)	14.06 (2.23)	14.54 (2.91)	13.90 (2.88)	14.30 (2.63)
Strategic approach to studying		74.72 (10.64)	70.42 (9.69)	71.13 (10.00)	70.78 (10.46)	72.91 (10.51)
	Organised study	14.35 (3.06)	13.39 (2.53)	13.03 (2.90)	12.46 (2.97)	13.73 (3.02)
	Time management	14.42 (3.38)	12.89 (3.57)	12.83 (3.04)	13.25 (3.64)	13.72 (3.44)
	Alertness to assessment demands	15.23 (2.51)	14.44 (2.50)	15.04 (2.71)	14.34 (2.45)	14.98 (2.57)
	Achieving	14.85 (2.71)	14.35 (2.44)	14.34 (2.68)	15.42 (2.43)	14.71 (2.65)
	Monitoring effectiveness	15.86 (2.41)	15.14 (2.00)	15.97 (2.33)	15.30 (2.08)	15.72 (2.32)
Surface approach to studying		48.39 (7.61)	52.49 (9.00)	48.02 (8.74)	49.72 (8.78)	49.08 (8.32)
	Lack of purpose	8.42 (3.27)	11.41 (3.47)	8.85 (3.07)	8.49 (3.65)	8.98 (3.46)
	Unrelated memorizing	11.84 (2.52)	12.59 (2.68)	11.67 (2.85)	11.96 (2.50)	11.93 (2.63)
	Syllabus-bound	13.81 (2.68)	13.95 (2.84)	13.53 (2.91)	14.25 (2.79)	13.81 (2.77)
	Fear of failure	14.32 (3.20)	14.54 (2.97)	14.34 (3.67)	15.01 (3.09)	14.43 (3.26)

*ASSIST* Approaches and Study Skills Inventory for Students, *M* Mean, *SD* Standard Deviation

### Predictors of academic performance

Being of higher age, being female, and spending more time on self-study activities were all directly associated with higher GPA. Among the deep approach subscales, higher scores on ‘seeking meaning’ showed a statistically significant association with higher GPA. Among the strategic approach subscales, lower scores on ‘time management’ and higher scores on ‘achieving’ were directly associated with higher GPA. Among the surface approach subscales, higher scores on ‘lack of purpose’ and lower scores on ‘fear of failure’ were directly associated with higher GPA. The strongest associations with GPA were shown for ‘achieving’ (std. *β* = 0.22, in the strategic category) and ‘fear of failure’ (std. *β* = −0.17, in the surface category). The full regression model explained 9.6% of the total variance in GPA among the students, and 7.0% of the GPA variance was explained by the ASSIST subscales. The results from the regression analysis are displayed in Table 3.

**Table 3:** Predictors of Grade Point Average among the participants (*n* = 712)

Independent variables	Grade Point Average	
1) Demographics and education	Std. *β*	*p*
Age	0.11	<0.01
Gender	−0.10	0.01
Prior higher education	0.01	0.79
Time spent on self-study	0.10	<0.01
Explained variance	2.6%	<0.01
2) Deep approach subscales		
Seeking meaning	0.12	0.01
Relating ideas	−0.05	0.30
Use of evidence	0.02	0.69
Interest in ideas	−0.08	0.08
R^2^ change	1.2%	0.08
Explained variance	3.8%	<0.01
3) Strategic approach subscales		
Organized study	−0.00	0.99
Time management	−0.12	0.04
Alertness to assessment	−0.06	0.17
Achieving	0.22	<0.001
Monitoring effectiveness	0.04	0.39
R^2^ change	1.8%	0.03
Explained variance	5.6%	<0.001
4) Surface approach subscales		
Lack of purpose	0.14	<0.01
Unrelated memorizing	−0.02	0.70
Syllabus-bound	−0.01	0.88
Fear of failure	−0.17	<0.001
R^2^ change	4.0%	<0.001
Explained variance	9.6%	<0.001

Table content is standardized *β* weights, indicating the strength of each variable’s relationship with GPA controlling for all variables in the model, and *p*-values associated with these relationships. Variable coding: female = 1, male = 2; prior higher education = 1, no prior higher education = 2. On all other variables, higher scores indicate higher levels

## Discussion

The aim of the current study was to examine associations between demographic background, education-related factors, approaches to studying, and academic performance among occupational therapy undergraduate students. A large cross-cultural sample was used, consisting of students from four different countries. The results indicate that higher age, female gender, and more time spent engaged in self-study were independently associated with higher GPA in the sample. Five of the 13 ASSIST subscales (higher ‘seeking meaning’, ‘achieving’ and ‘lack of purpose’, and lower ‘time management’ and ‘fear of failure’) were also independently associated with higher GPA. Thus, some of the associations between subscale scores and GPA - but not all - were in the hypothesized direction.

### Demographic and education-related predictors of GPA

In line with previous research [18, 42], being older and being female were associated with higher GPA in the sample. These results remained even after controlling for the ASSIST subscale scores. Thus, positive effects of being older and being female are not fully explained by more productive approaches to studying among older and female students. In spite of relatively small effect sizes, being older and being female appears to be of importance for the students’ academic performance in ways that extend the older and female students’ perhaps more productive approaches to studying.

Similarly, there remained a significant effect of time spent engaged in self-study even when controlling for the ASSIST subscale scores. Thus, it appears that positive learning outcomes, as operationalized in higher GPA, is not *all* about how the students engage with content: a purely quantitative measure of time spent engaging with study content is important in and of itself. In popular terms, it does not all come down to how you study, but studying, regardless of how, counts as well. To an extent, then, all students, regardless of their motivational type and their ways of engaging with studying, can improve their academic performance by spending more time with study-related materials and tasks. Recent research demonstrated that time spent engaged in self-study also can predict higher satisfaction with students’ selected programmes of study [14]. Taken together, these results suggest that there may be several valid reasons for students to spend more time engaging with their studies.

### Predictors of GPA among the ASSIST subscales

As noted, spending time with study materials and tasks are important in and of itself, but it is also a matter of how the time is spent. This study showed that higher scores on the ASSIST ‘seeking meaning’ subscale were associated with higher GPA among the students. Thus, this aspect of the ASSIST’s deep approach to studying seems to be particularly important for learning outcomes. The student who seeks meaning generally wants to find out and reflect on the new information they are exposed to and what it means in relation to what he or she already knows, and seeks to contrast or expand knowledge by connecting it to situations where it can be applied [8]. The student’s motivation is to increase his or her own understanding. This way of relating to academic and course content is logically related to improved learning outcomes in higher education, and the finding builds from and expands on previous research in the field that has emphasized the importance of a deep learning approach [17, 18, 20, 23, 43].

The strategic approach subscale ‘Achieving’ was the strongest predictor of students’ higher GPA. Previous research has similarly emphasized the strategic approach to studying as a productive one [18, 24], and the current study suggests that the ASSIST ‘achieving’ subscale may be of particular importance. The achievement-oriented student places much effort on studies and is strongly motivated to do what it takes to get good grades. Thus, the association between higher scores on the ASSIST ‘achieving’ subscale and higher GPA is logical. In fact, the combination of a strong motivation for getting good grades (achieving), allocating much time dedicated to self-study (time spent on self-study activities), and studying with the main purpose of understanding (‘seeking meaning’) may be particularly helpful for occupational therapy students seeking success with their academic studies.

Related to the surface approach, the ASSIST ‘fear of failure’ subscale predicted lower GPA, and this is similarly consistent with previous findings in the field [13, 18, 20]. The student fearing failure often feels overwhelmed with the amount of study materials, worries if he or she is going to make it, and can start panicking if feeling behind with the work. It appears logical that such an anxiety-laden approach to studying does not translate into good results. Students’ energy and attention directed towards their fear of potentially failing mean less mental focus being available for actual studying.

This analytic dichotomy, fear of failure versus desire for achievement, has been applied in a range of fields, and has been associated with individual as well as collective performance [44]. We may think of a person who is about to give an important speech, or a sports team scheduled to play against another competitor. One anecdote seems timely: Just a few days before writing these lines, Iceland’s national football team defeated England’s by 2–1 in a game during the European Championship in France. Iceland’s population is about 330,000 [45], whereas England’s is more than 65 million [46]. We can only speculate, but we would not be surprised if it was found that the Icelandic team was strongly motivated by a desire to win the game (‘achieving’), while England’s team was more concerned with avoiding losing it (‘fear of failure’). Obviously, however, many other factors may have influenced this outcome.

Somewhat surprisingly, we found that lower scores on ‘time management’ (strategic approach) was associated with higher GPA among the students. This finding appears to contradict the theory and some of the research related to the ASSIST scales [3–5, 7]. It is possible, though, that some of the highly organized students with presumably high scores on ‘time management’ are less concerned with the actual content of their study, and may be less concerned with how their studying assists their actual learning. This is a picture of a somewhat ritualistic student: the student performs study activities as expected, but may care less about what it means and how he or she can make use of it later.

Contrastingly, some of the less organized students may well be more prone to examine the meaning of the study content, and may more often try to examine and relate different ideas. Study efforts in terms of time use, however, may vary considerably from day to day. In short, these students may have more in common with the deep learner prototype [8, 32]. Therefore, they may learn much from their (somewhat unstructured) studies, and get good grades as a result.

In the current study, we also found that higher scores on ‘lack of purpose’ (surface approach) was associated with higher GPA. This may be even more of a surprise, as it is in direct contrast to previous research findings concerned with the negative impact from a surface approach to studying [18, 24, 47]. However, considering the items belonging to the scale (items expressing that the study may not be worthwhile, interesting, or relevant), one possible explanation may be that some of the very capable students consider the occupational therapy education too easy, in that it presents them with not enough of an intellectual challenge. If this were the case, these students would have rated the ASSIST ‘lack of purpose’ subscale at a high level, while still performing well on the exams. One should also take into account previous criticisms of the theoretical model underpinning the ASSIST [48], suggesting that the deep versus surface learning dichotomy is overly simplistic. In other words, the best-suited approach to studying may not be the same regardless of context, but it may depend on the nature of the knowledge to be acquired. A recent study of first year undergraduate occupational therapy students in Israel found that students who participated in the out-of-class training showed significant increases in their knowledge and competence, and they had better grades when compared with students who received regular activity analysis training in courses that required knowledge of accessibility [49]. This shows that the teaching approach or strategy, and not solely the students’ attitudes, might contribute to enhance students’ awareness and interest in studying.

### Study limitations

The study has several limitations. Given that we have presented results from a cross-cultural study, the relatively small number of students recruited from Singapore and the substantially larger number of students from Australia represents one limitation. In addition, only first year students were included in the Singapore, and the Hong Kong sample included only five students from the second year of their occupational therapy study. The limitations of the study further included the use of a convenience sampling approach and self-report questionnaires, both of which can lead to bias in the results. The study also does not take into account other factors that may be associated with the adopted study approaches, such as students’ perception of workload in their academic course [50], types of assessments conducted and also the teaching pedagogy of the curriculum in four different programs.

### Future research

A similar study could be completed with a larger number of student groups from a larger number of countries. The approaches to study of undergraduate occupational therapy students could be compared to graduate-entry masters or entry-to-practice clinical doctorate occupational therapy students. Occupational therapy students’ approaches to study could also be compared to other health professional student groups to examine if similarities or differences exist. Moreover, perhaps with particular relevance for a practice-based and skills-oriented profession like occupational therapy, outcome variables in future studies may go beyond the commonly used GPA as a measure of academic performance. Becoming a competent and effective occupational therapist takes more than good academic grades, a perspective that preferably should be reflected in future research on occupational therapy students.

## Conclusions

This study found that higher age, female gender, and time spent self-studying all predicted higher GPA in a cross-cultural sample of undergraduate occupational therapy students. Thus, educators’ advice to students about spending more time involved in self-study activities is warranted. Five of the ASSIST subscales also predicted higher GPA in the students. Based on the results, educators should teach and advise students about using productive study approaches when engaging with course and subject content. Educators should also be careful to employ teaching strategies that facilitate and promote students to engage in the learning process using deep learning strategies such as searching for meaning and relationships in the study curriculum [51].

The message to students is that they should seek meaning (study in order to understand more fully) and orient themselves toward achievement (study in order to do their best), rather than trying to avoid failure. The fear of failure approach appears to have a doubly unproductive impact: i) it gives the student a lot to worry about, and ii) it leads to poorer academic results. The small effect sizes obtained from the study suggests that researchers should continue to investigate predictors of academic achievement among occupational therapy students.
